# Dimethylaminoparthenolide (DMAPT) as an alternative approach for treatment of Familial Mediterranean Fever (FMF)

**DOI:** 10.22038/IJBMS.2021.59180.13140

**Published:** 2021-10

**Authors:** Ali Mosayebian, Roya Sherkat, Saied Abediankenari, Monireh Golpour, Alireza Rafiei

**Affiliations:** 1 Department of Immunology, School of Medicine, Mazandaran University of Medical Sciences, Sari, Iran; 2 Acquired Immunodeficiency Research Center, Isfahan University of Medical Sciences, Isfahan, Iran; 3 Immunogenetics Research Center, Faculty of Medicine, Mazandaran University of medical sciences, Sari, Iran; 4 Molecular and Cell Biology Research Center, Student Research Committee, Faculty of Medicine, Mazandaran University of Medical Science, Sari, Iran

**Keywords:** CASP1, Dimethylamino-arthenolide, Familial Mediterranean – Fever, IL-1β, IL-18, MEFV, NFκB, NLRP3

## Abstract

**Objective(s):**

Familial Mediterranean Fever (FMF) is a hereditary auto-inflammatory disorder that is caused by mutations in the Mediterranean fever (MEFV) gene and is associated with an increase in pro-inflammatory cytokines, such as interleukin-1β (IL-1β) and interleukin-18 (IL-18), leading to excess inﬂammation. Colchicine is a common drug widely used for treatment of FMF attacks, but about 5–15% of the patients show resistance to the regular colchicine treatment. In this study, we used dimethylamino-parthenolide (DMAPT), as a small molecule inhibitor of Nuclear factor-κB (NF-κB), NLR family Pyrin domain containing 3 (NLRP3), and cysteine-aspartic acid protease 1(Caspase-1) on FMF-derived peripheral blood mononuclear cells (PBMCs).

**Materials and Methods:**

The effects of DMAPT and colchicine on metabolic activity and apoptosis of FMF-derived PBMCs were evaluated by MTT and Annexin V/PI assays, respectively. Also, the expression levels of NF-κB, NLRP3, MEFV, CASP1, and IL-1β mRNA were investigated using a TaqMan real-time PCR, and the protein levels of IL-1β, IL-18, and IL-37 were assessed via an enzyme-linked immunosorbent assay (ELISA) in LPS/ ATP-stimulated PBMCs.

**Results:**

DMAPT decreased the expression levels of NFκB (0.38±0.096, *P<*0.0001), NLRP3 (0.39±0.12, *P<*0.001), MEFV (0.384±0.145, *P<*0.001), CASP1 (0.48±0.13, *P=*0.0023), and IL-1β (0.09±0.09, *P<*0.0001) and reduced the secretion levels of IL-1β (8.92±5.3 vs. 149.85±20.92, *P<*0.0001), IL-18 (135±32.1 vs. 192±22.18, *P=*0.01), and IL-37 (27.5±6.3 vs. 78.19±14.3, *P<*0.0001) as compared to untreated cells.

**Conclusion:**

Given the obtained results in comparison with previous research, the future clinical development of DMAPT could result in the expansion of new anti-inflammatory therapeutics for FMF disorder.

## Introduction

Autoinflammatory disorders (AIDs) or periodic fever syndromes are a group of hereditable disorders characterized by recurrent episodes of fever and severe inflammation, most commonly in the gut, skin, joints, and eyes. These are monogenic or polygenic diseases and include familial Mediterranean fever (FMF), TNF receptor-associated periodic syndrome (TRAPS), hyperimmunoglobulinemia D with periodic fever syndrome (HIDS), cryopyrin-associated periodic syndrome (CAPS), Majeed syndrome, Blau’s syndrome, aphthous stomatitis, pharyngitis, and adenitis syndrome (PFAPA), deficiency of interleukin-1 receptor antagonist (DIRA), deficiency of IL-36 receptor antagonist, pyogenic arthritis, pyodermagangrenosum and acne syndrome (PAPA), Crohn’s disease, Still’s disease, and Behçet’s disease. In addition, acquired autoinflammatory syndromes such as Schnitzler’s syndrome are also classified as multifactorial autoinflammatory diseases (1). The prevalence of autoinflammatory disorders in the Iranian population is more than 1/10,000 and the expected frequency of patients with these disorders would be approximately 8000 individuals (2).

FMF is a hereditary auto-inflammatory disorder characterized by recurrent fever and abdominal pain with serositis, joint pain, and skin lesions. FMF mainly affects the Mediterranean and Middle Eastern people, such as Turks, Arabs, Jews, Armenians, Cypriots, Italians, and Spaniards. However, some FMF cases have been reported in other ethnic groups living far from the Mediterranean ancestry. It is estimated that about 150,000 people worldwide have the disease (3, 4).

This disease is an autosomal recessive disorder, but there is also an autosomal dominant form of it caused by heterozygous pathogenic variants in the Mediterranean fever (MEFV) gene (5-7). The MEFV gene encodes Pyrin which is a 781-amino acid protein that forms an element of the NLRP3 and Pyrininﬂammasome complex, regulates interleukin-1β (IL-1β) processing, and plays an important role in the control of inflammation and innate immunity. Mutations in the MEFVgene are associated with an increase in IL-1β level giving rise to an excess inﬂammation (8). Therefore, controlling the function of the inﬂammasome complex plays an effective role in inhibiting the corresponding inflammation as well as the production of IL-1β and IL -18 cytokines.

Colchicine is a microtubule-depolymerizing drug widely used for the treatment of gout and arthritis and the prevention of FMF attacks and secondary amyloidosis development. Although colchicine is known as an effective drug in preventing FMF attacks, about 5–15% of FMF patients show resistance to a regular colchicine treatment and thus experience recurrent attacks. Recently, these patients have been treated with IL-1 inhibitors, such as Canakinumab, Anakinra, and Rilonacept to suppress auto-inflammation (9-11). Yet there are some disadvantages associated with these biologic drugs including high cost, severe reactions in the injection site, and inability to target intracellular mediators (12, 13).

One strategy to overcome the mentioned drawbacks of biological drugs could be using small molecules (SMs) which could inhibit inflammation and production of inflammatory cytokines by innate immune cells. One of these SMs is parthenolide, which is a sesquiterpene lactone extracted from the Feverfew plant or *Tanacetumparthenium* that is a medicinal plant traditionally used for treatment of fevers, migraine headaches, rheumatoid arthritis, stomach aches, toothaches, insect bites, infertility, and menstrual problem. The Feverfew herb has a long history of application in traditional and folk medicine, especially by Greek and early European herbalists. This plant contains a large number of natural products, such as sesquiterpene lactones (parthenolide) (14). Several studies have already illustrated the anti-inflammatory and anti-tumor effects of parthenolide(PTL) and its derivatives which inhibit the activity of Nuclear factor-κB (NF-κB), NLR family Pyrin domain containing 3 (NLRP3), and cysteine-aspartic acid protease 1(Caspase1) (15, 16). 

In this study, dimethylaminopartenolide (DMAPT) was applied as one of the PTL derivatives with a bioavailability of 70% stemmed from its significant water solubility which is more than 1000 times higher relative to PTL with a bioavailability of 2% (17). And this study aimed to investigate the anti-inflammatory effect of DMAPT on peripheral blood mononuclear cells (PBMCs) of FMF patients in comparison with colchicine (the positive control). 

## Materials and Methods

***Study population***

A total of 8 FMF patients who were admitted to Alzahra Teaching Hospital, Isfahan, Iran, participated in this study. FMF was diagnosed based on Tel Hashomer and Turkish FMF pediatric clinical criteria, and the MEFV gene’s pathogenic variant and mutation were confirmed by the whole-exome sequencing (WES). Clinical, demographic, and MEFV gene variants of FMF patients were also assessed (Table1). Written informed consent was obtained from all participants in accordance with the Declaration of Helsinki, and the study protocol was approved by the Ethics Committee of the Mazandaran University of Medical Sciences (IR.MAZUMS.REC.1398.079). 

***Isolation and culture of PBMCs***

Peripheral blood samples were collected from FMF patients, and PBMCs were immediately isolated by density gradient centrifugation using Ficoll-Hypaque (Biochrom, Berlin, Germany). Trepan blue dye exclusion test revealed the viability of the isolated cells to be more than 98%. PBMCs were cultured in a Roswell Park Memorial Institute (RPMI) 1640 culture medium (Hyclone, Logan, Utah) complemented with 10% fetal bovine serum (FBS) and 1% penicillin/streptomycin using 24-well plates at a density of 1×10^6^ cells per well and 96-well plates at a density of 2×10^5^ cells per well for 24 hr. Afterward, PBMCs were treated for the next 24 hr with various concentrations (i.e., 6.25, 12.5, 25, and 50 µM) of DMAPT (ab146189, Abcam, Cambridge, United Kingdom) or colchicine (Sigma-Aldrich, Missouri, USA). The cells were incubated at 37 ^°^C under a humidified atmosphere containing 5% CO_2_.

***Metabolic activity of PBMCs***

MTT (3-[4,5-dimethylthiazol-2-yl]-2, 5-diphenyl tetrazolium bromide) assay was applied to evaluate cell proliferation/cytotoxicity activity resulting from treating PBMCs with DMAPT and colchicine. Briefly, 20 µl of MTT (Sigma-Aldrich, Missouri, USA) (5 mg/ml) was added into each well, and the plate was then incubated for 4 hr at 37 ^°^C under a humidified atmosphere containing 5% CO_2_. Next, the plate was centrifuged at 300 g for 10 min, and 100 µl of dimethyl sulfoxide (DMSO) (Sigma-Aldrich, Missouri, USA) was added to the wells after removing the previous medium. The optical density was measured using an ELISA plate reader (Synergy H1 BioTek, Winooski, USA) at 570 nm. Results were expressed as a percentage of viability, and 100% represented the control cells treated with 0.1% DMSO alone. Moreover, the half-maximal inhibitory concentration (IC50) value was calculated for DMAPT and colchicine for further investigations.

***Real-time PCR for gene expression analysis***

Total RNA was collected using a Hybrid-R blood RNA mini protocol (Geneall, Seoul, Korea) from 10^6^ PBMCs treated with DMAPT (10 μM) or colchicine (10 μM). The quantity and quality of the extracted RNA were assessed through the absorbance ratio (A260/A280) and electrophoresis on the agarose gel. The collected total RNA was kept at −70 ^°^C to be used for further assays.

The expression levels of NFκB, NLRP3, MEFV, Caspase1, and IL-1β mRNA were quantified via a unique sequence index (USI) barcode and a TaqMan probe proposed by Fattahi *et al*. (18). Also, the GAPDH gene was used as an endogenous housekeeping control. All sample analyses were performed in triplicate, and specific gene primers were developed using the AlleleID 6.0 software package (Table 2).

To this end, RNA samples were transcribed into cDNA via mRNA-specific USI RT-PCR primers and Addscript RT master (Addscript RT Master, Addbio, Korea). For cDNA synthesis, the reaction was firstly incubated at 25 ^°^C for 10 min to expand the primers before increasing the reaction temperature to 42 ^°^C for 60 min, then the reaction was completed by incubation at 70 ^°^C for 10 min. Gene expression analysis was performed using Rotor-Gene Q (QIAGEN, Hilden, Germany), and Hot StarTaqPlus DNA Polymerase (QIAGEN, Hilden, Germany) was utilized for the real-time PCR reaction. The amplification protocol consisted of an initial denaturation at 95 ^°^C for 5 min followed by 45 cycles consisting of denaturation at 95 ^°^C for 45 sec and annealing at 60 ^°^C for 1 min. Furthermore, gene quantification was calculated via the Livak method.

***Measurement of cytokine concentration***

In order to investigate cytokine production, PBMCs were treated with DMAPT or colchicine (10 µM) at 37 ^°^C for 24 hr in an RPMI 1640 medium containing 5% CO_2_; then, they were stimulated by 1 μg/ml of LPS (originated from Escherichia coli serotype B4/L4391) for 3 hr to activate the classical NF-κB pathway or by LPS for 3 hr and ATP (5 mM) for an additional 30 min (LPS/ATP) to activate the NLRP3 inflammasome. Both LPS and ATP were obtained from Sigma-Aldrich (Sigma-Aldrich, Missouri, USA). The cell supernatants were collected and stored at -20 ^°^C for future analysis. IL-1β, IL-18, and IL-37 levels were measured in the culture supernatant using commercially available enzyme-linked immunosorbent assay kits (R&D Systems, MN, USA) according to the manufacturer’s recommendations. The minimum detectable concentrations of IL-1β, IL-18, and IL-37 were determined as 3.9, 26.6, and 15.6 pg/ml, respectively. All sample analyses were performed in duplicate.

***Flow cytometric determination of apoptosis***

Annexin V-FITC and PI labeled Apoptosis Assay kit (eBioscience, San Diego, USA) was used to assess apoptosis according to the manufacturer’s protocol. In brief, PBMCs (1×10^6^ cells/ml) were placed into 24-well plates, and after treatment for 24 hr with different concentrations of DMAPT or Colchicine (i.e., 6.25, 12.5, 25, and 50 µM) as well as DMSO 5% (the positive control), the cells were harvested and washed by a cold phosphate-buffered saline (PBS) solution. The cells were resuspended using a staining buffer, and 5 µl of Annexin V-FITC along with 5 µl of PI were added to stain the cells. All samples were mixed gently and incubated for 15 min in the dark at room temperature. Samples were then assayed within 1 hr by a FACSCalibur flow cytometer (Becton Dickinson, San Jose, CA, USA), and the obtained data were analyzed using the FlowJo software package (v10.1, FlowJo, Ashland, OR, USA).

***Statistical analysis***

Normal distribution of data was tested with the Kolmogorov–Smirnov test. Depending on the type of distribution and the quantity or quality of the variable, parametric tests (such as t-test and analysis of variance) and non-parametric tests (such as Mann-Whitney and Cross-call-Wallis) were used. The quantitative data were presented as mean±standard error (SE) of the mean. *P*-value less than 0.05 was considered to indicate statistical significance.

## Results

***Demographic and clinical characteristics of FMF patients***

Two out of 8 patients were female, and the male to female ratio was 4:1. Their mean age at the last visit was 24.37±14.76 years old at the range of 10-58, and 62.5% of patients had family relationships. The duration of the disease was 7.1±6.1 years, and 75% of patients were genetically diagnosed. The percentages of homozygote, heterozygote, compound heterozygote, and unknown mutations were 50, 25, 12.5, and 12.5, respectively. The most important clinical manifestations in patients were fever (100%), abdominal pain (100%), fatigue (87.5%), myalgia (87.5%), headache (62.5%), and chest pain (62.5%)**.** Fever duration ranged from 1 to 7 days with a frequency of every 15 days to every 60 days. All patients were treated with colchicine, and to eliminate the interfering factors, treatment stopped 48 hr before sampling.

***DMAPT decreased the proliferation rate of FMF-derived PBMCs in a dose-dependent manner***

**Figure 1:**
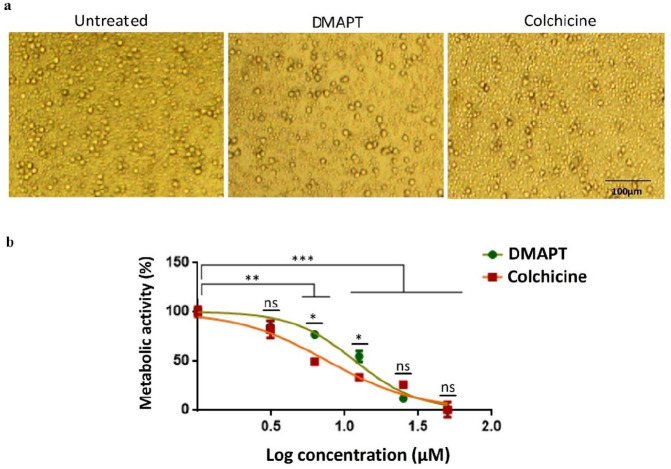
Comparison of anti-proliferative effects of Dimethylamino-parthenolide (DMAPT) and colchicine on the viability of Familial Mediterranean Fever (FMF) derived PBMCs. Microscopic images of untreated and DMAPT or colchicine treated cells (a). Percentage of the metabolic activity of untreated and treated PBMCs at different doses of DMAPT or colchicine (3.125, 6.25, 12.5, 25, and 50 µM) after 24 hr obtained by the MTT assay (b). The experiments were performed in triplicate, and the data were reported as means±SD (ns= non-significant, ^*^P<0.05, ^**^P<0.01, ^***^P<0.001)

As shown in the microscopic image of PBMC cells (Figure 1A), the proliferation of DMAPT (10 μM) treated cells reduced as compared with untreated cells, and it was comparable to the effect of colchicine (10 μM). The findings of the MTT assay conducted on treated PBMCs showed that DMAPT and colchicine prevented the proliferation of FMF-derived PBMCs in comparison with untreated cells in a dose-dependent manner over 24 hr, and there was a negative correlation between the obtained cell survival ratio and concentrations of DMAPT and colchicine (Figure 1B). The metabolic activity of PBMCs treated with 3.125, 6.25, 12.5, 25, and 50 μM of DMAPT was 84.5, 76.5, 54.58, 17.66, and 4.25, respectively. The calculated IC50 values (95% confidence interval) of DMAPT and colchicine after 24 hr were 11.73 (10.09 to 13.64) and 9.1 (7.38 to 10.91) μM, respectively. Therefore, the concentration of 10 μM was selected for DMAPT and colchicine for further investigations which was in line with previous reports (19). 

***DMAPT*** ***reduced LPS/ATP-induced NF-ĸB mRNA expression***

**Figure 2:**
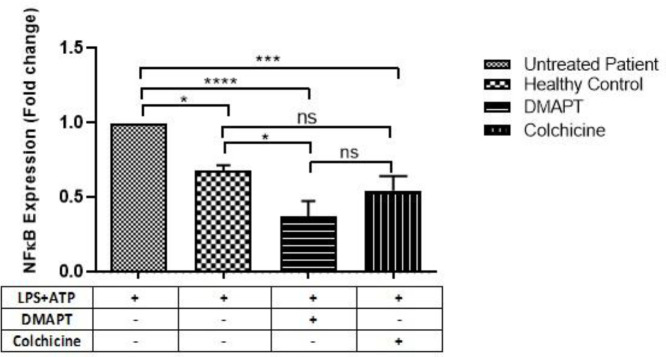
Effects of DMAPT and colchicine on the NF-ĸB expression in PBMCs stimulated by LPS and ATP. DMAPT inhibited LPS/ATP-induced mRNA expression of NF-ĸB in PBMCs. PBMCs (1.5×10^6^) were treated with DMAPT or colchicine (10 µM) for 24 hr and then stimulated by LPS (1 µg/ml) for 3 h and ATP (5 mM) for an additional 30 min. Total RNA extraction was performed as described in Materials and Methods. The mRNA expression levels were calculated based on the ratio of treated PBMCs to untreated PBMCs and were normalized to the expression levels of GAPDH. The data were reported as mean±SE of three independent experiments (^*^P<0.05, ^**^P<0.01, ^***^P<0.001)

As shown in Figure 2, DMAPT significantly reduced LPS/ATP-induced NF-κB expression relative to control (0.38±0.096, *P<*0.0001), and a similar effect was also observed for colchicine (0.55±0.12, *P=*0.0009). Furthermore, DMAPT exhibited a greater inhibitory impact on the gene expression of NF-κB as compared with colchicine, albeit it was not significant. Also, in all assays carried out to evaluate gene expression and cytokine production, healthy untreated control samples were used, and the stimulatory effects of LPS and ATP on them were compared with those on untreated cells collected from patients. As a result, LPS/ATP significantly increased NF-κB expression relative to the healthy control (0.68±0.096, *P=*0.012).

***DMAPT*** ***reduced LPS/ATP-induced NLRP3, MEFV, Caspase1, and IL-1βmRNA expressions in PBMCs***

**Figure 3:**
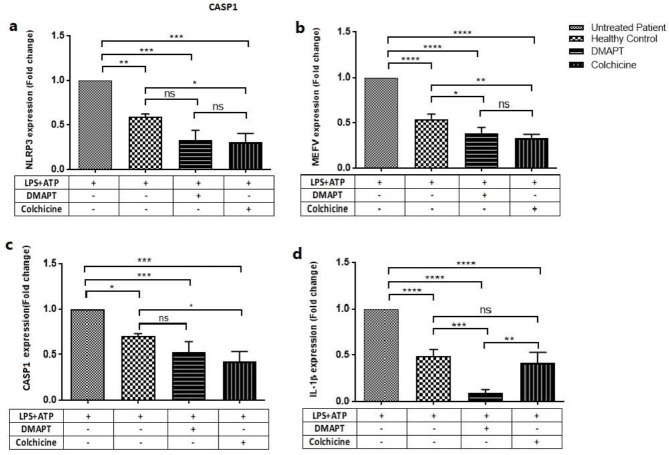
Effects of DMAPT and colchicine on NLRP3, MEFV, CASP1, and IL-1β expression levels in PBMCs stimulated by LPS and ATP. DMAPT and colchicine reduced LPS/ATP-induced NLRP3 (a), MEFV (b), CASP1 (c), and IL-1β (d) expression levels in PBMCs. The mRNA expression of NLRP3, MEFV, CASP1, and IL-1β was quantified by TaqMan real-time PCR and normalized to the expression levels of GAPDH. The altered gene expression was shown as fold changes referring to untreated PBMCs of the patient. The data were reported as mean±SE of three independent experiments (^*^P<0.05, ^**^P<0.01, ^***^P<0.001, ^****^P<0.0001)

Since DMAPT and colchicine inhibit NF-κB expression, they may also affect the expression of other NLRP3-inflammasome components, such as NLRP3, ASC, and Caspase1, and pro-inflammatory cytokines, such as IL-1β and IL-18. Therefore, we examined the expression levels of NLRP3, MEFV, CASP1, and IL-1β in the presence and absence of DMAPT or Colchicine. In this regard, FMF-derived PBMCs were treated with DMAPT or colchicine (10 µM) for 24 hr and then stimulated by LPS (1 µg/ml) for 3 hr and ATP (5 mM) for 0.5 hr. As depicted in Figure 3, compared with untreated FMF-derived PBMCs, DMAPT and colchicine significantly reduced LPS/ATP-induced NLRP3 (0.39±0.12, *P<*0.001 and 0.31±0.12, *P<*0.001), MEFV (0.384±0.145, *P<*0.001 and 0.332±0.089, *P<*0.001), CASP1(0.48±0.13, *P=*0.0023 and 0.37±0.13, *P=*0.002), and IL-1β (0.09±0.09, *P<*0.0001 and 0.42±0.09, *P=*0.002) mRNA expressions. Also, DMAPT significantly decreased IL-1β expression in comparison with colchicine (0.09±0.09 vs. 0.42±0.09, respectively *P=*0.0043) (Figure 3). By comparing the results obtained for untreated PBMCs of FMF patients and healthy controls, a significant increase was observed in the expression of NLRP3 (0.40±0.098, *P=*0.0013), MEFV (0.45±0.053, *P<*0.0001), CASP1 (0.59±0.09, *P=*0.0137), and IL-1β (0.45±0.09, *P<*0.0001).

***DMAPT*** ***suppressed the secretion levels of IL-1β, IL-18, and IL-37 in LPS/ATP-induced PBMCs***

**Figure 4:**
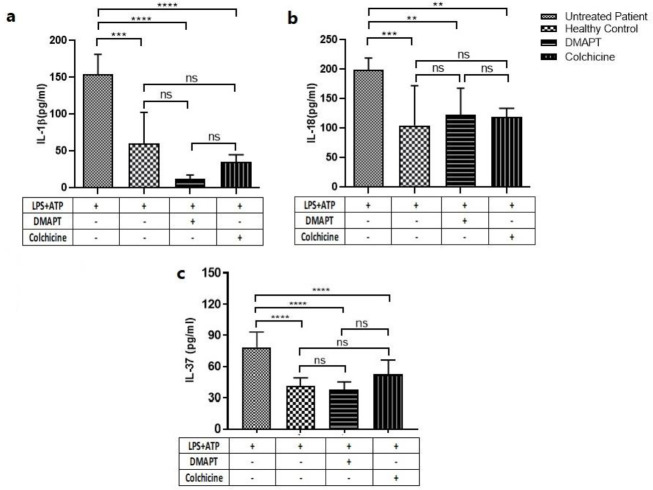
Effects of DMAPT and colchicine on IL-1β, IL-18, and IL-37 levels in PBMCs stimulated by LPS and ATP. DMAPT and colchicine decreased the levels of IL-1β (a), IL-18(b), and IL-37(c). The data were reported as mean±SE of three independent experiments (^*^P<0.05, ^**^P<0.01,^ ***^P<0.001, ^****^P<0.0001)

To evaluate the effect of DMAPT on the inflammasome-specific cytokine secretion, the secretion levels of IL-1β, IL-18, and IL-37 were measured in the cell culture supernatants of LPS/ATP-induced PBMCs by ELISA. As shown in Figure 4, DMAPT significantly reduced secretion levels of IL-1β (8.92±5.3 vs. 149.85±20.92, *P<*0.0001), IL-18 (135±32.1 vs. 192±22.18, *P=*0.01) and IL-37 (27.5±6.3 vs. 78.19±14.3, *P<*0.0001) in PBMCs of FMF patients as compared to untreated cells, respectively. 

It was also found that the levels of IL-1β (149.85±20.92 vs.55.5±37.5, *P=*0.0005), IL-18 (192±22.18 vs.102±57.4, *P=*0.0009), and IL-37 (78.19±14.3 vs.36.69±5.80, *P<*0.0001) in the untreated PBMCs of FMF patients were significantly higher than untreated healthy controls.

***DMAPT increased apoptosis levels in FMF-PBMCs***

**Figure 5:**
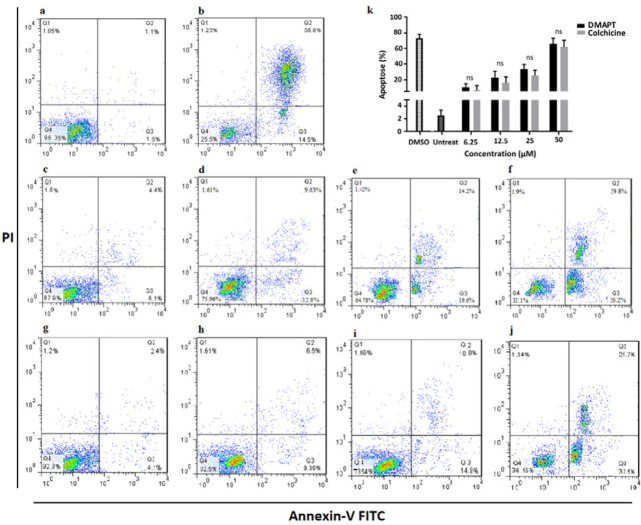
AnnexinV/PI double-staining analysis of FMF-derived PBMCs treated by various concentrations DMAPT or colchicine. The cells were stained with annexin V-FITC and PI and analyzed by flowcytometry. Negative (a) and positive (treated by DMSO 5%) (b) controls. Different concentrations of DMAPT (6.25, 12.5, 25 and 50 µmol/l) (c-f). Different concentrations of colchicine (6.25, 12.5, 25, and 50 µmol/l) (g-j). Comparative effects of different concentrations of DMAPT as well as colchicine on apoptosis of treated PBMCs (k).Quantification of apoptotic cells (%) revealed DAMPT has a similar impact on PBMCs as compared to Colchicine and no significant difference were observed between DMAPT and Colchicine in each dose. Data are presented as the percentage of apoptotic cells (ns=non-significant)

To assess the effect of DMAPT or colchicine on the cell death pattern and apoptosis in FMF-derived PBMCs, the Annexin-V/PI assay was employed. As shown in Figure 5, the apoptosis levels in PBMCs were related to the concentration of DMAPT or colchicine. Hence, the proportions of apoptotic cells treated with 6.25, 12.5, 25, and 50 µmole/l of DMAPT for 24 hr were 10.5, 22.43, 33.8, and 66 %, respectively; also, they were respectively 6.5, 15.89, 25.6, and 62.2 % for the same corresponding concentrations of colchicine after 24 hr of treatment**.** No significant difference was observed between DMAPT and colchicine in any treatment doses. Flow cytometry analysis was performed according to standard protocol. On the other hand, in addition to isotype controls, we used untreated PBMCs as the negative control, 5% DMSO treated PBMCs as the positive control, and untreated and unstained PBMCs for eliminating of autofluorescence background.

**Table 1:** Clinical, demographic, pathogenic variant, and mutation type of Familial Mediterranean Fever (FMF) patient

**Cases**	**P1**	**P2**	**P3**	**P4**	**P5**	**P6**	**P7**	**P8**
MEFV genotype	Homozygote	Homozygote	Heterozygote	Homozygote	Compound heterozygote	Homozygote	Unknown	Unknown
Genetic mutation	M694I/M694I	M694I/M694I	M694I/Wild type	E148Q/E148Q	M680I/R761H	M694V/M694V	_	_
Gender (F/M)	M	M	F	M	M	M	M	M
Age at last visit	27	34	58	22	18	12	10	14
Age at onset (years)	26	25	40	20	2.5	6	8	11
Disease duration (years)	1	9	18	2	15.5	6	2	3
Fever	+	+	+	+	+	+	+	+
Fever duration	1-3	1-3	1-3	2-3	1-3	3-7	1-3	3-7
Fever frequency	Every 15 days	Every 15-30 days	Every 60 days	Every 30 days	Every 60 days	Every 15 days	Every 15 days	Every 30 days
Fatigue	+	+	+	_	+	+	+	+
Myalgia	+	+	+-	_	+	+	+	+
Headache	+	+	+	_	+	-	+	-
Abdominal pain	+	+	+	+	+	+	+	+
History of appendectomy	_	_	+	_	+	_	-	-
Chest pain	+	+	+	_	+	-	+	-
Acute phase reactant	+ (ESR, CRP, Ferritin, WBC)	+ (ESR, CRP, WBC, PLT)	+(ESR)	+ (ESR, CRP, PLT, Ferritin, WBC)	_	_	Acute-phase reactant	+ (ESR, CRP, Ferritin, WBC)
Response to colchicine	+	+	+	+	+	+	+	+

MEFV: Mediterranean fever, ESR: Erythrocyte sedimentation rate, CRP: C-reactive protein, WBC:With Blood Cell

**Table 2:** Primer sequences used for RNA quantification PCR

**Gene name**	**Accession number**	**Primers 5′ → 3**
**NF** **B**	NM_003998	Specific forward primer: GCCTGTCCTTTCTCATCCCATCT RT-PCR primer: GTCGTATCCAGTGCTGCGACCGTATGGATGTGTCTGCGGCGTTTTATCATGCACTGGATACGACGTCCTCTTTCTGC
**NLRP3**	NM_001079821.3	Specific forward primer: TCGGAGACAAGGGGATCAAACTAC RT-PCR primer: GTCGTATCCAGTGCTGCGACCGTATGGATGTGTCTGCGGCGTTTTATCATGCACTGGATACGACGCTGGAGGTCAG
***MEFV***	NM_001198536.2	Specific forward primer: CACTCTGCTGGTCACCTACTATGG RT-PCR primer: GTCGTATCCAGTGCTGCGACCGTATGGATGTGTCTGCGGCGTTTTATCATGCACTGGATACGACGCGGAATCATCTG
CASP1	NM_001257118.3	Specific forward primer: TGCAAGAATATGCCTGTTCCTGTG RT-PCR primer: GTCGTATCCAGTGCTGCGACCGTATGGATGTGTCTGCGGCGTTTTATCATGCACTGGATACGACACCATCTGGCTG
**IL-1β**	NM_000576.3	Specific forward primer: GGCTGCTCTGGGATTCTCTTC RT-PCR primer: TGTCGTATCCAGTGCTGCGACCGTATGGATGTGTCTGCGGCGTTTTATCATGCACTGGATACGACCATTGCCACTG
**GAPDH**	NM_001289745	Specific forward primer: TTGTCAAGCTCATTTCCTGGTATG RT-PCR primer: GTCGTATCCAGTGCTGCGACCGTATGGATGTGTCTGCGGCGTTTTTCATGCACTGGATACGACGGAGGCCATGTAG

NFκB: Nuclear factor- κB, NLRP3: NLR family Pyrin domain containing 3, MEFV: Mediterranean fever, CASP1: cysteine-aspartic acid protease 1, IL-1β: interleukin-1β, GAPDH: Glyceraldehyde 3-phosphate dehydrogenase

LPS: Lipopolysaccharide, ATP: Adenosine triphosphate, PBMCs: Peripheral blood mononuclear cells, DMAPT: Dimethylaminoparthenolide, NF-ĸB: Nuclear factor- κB, GAPDH: Glyceraldehyde 3-phosphate dehydrogenase

DMAPT: Dimethylaminoparthenolide, LPS: Lipopolysaccharide, NLRP3: NLR family Pyrin domain containing 3, MEFV: Mediterranean fever, CASP1: cysteine-aspartic acid protease 1, IL-1β: interleukin-1β, PBMCs: Peripheral blood mononuclear cells, ATP: Adenosine triphosphate, GAPDH: Glyceraldehyde 3-phosphate dehydrogenase

DMAPT :Dimethylaminoparthenolide, LPS: Lipopolysaccharide, ATP: Adenosine triphosphate, IL-1β: interleukin-1β, IL-18: interleukin-18, IL-37: interleukin-37, PBMCs: Peripheral blood mononuclear cells

PI: Propidium iodide, DMSO: Dimethyl sulfoxide, DMAPT: Dimethylaminoparthenolide, PBMCs: Peripheral blood mononuclear cells

## Discussion

Over-activation of NF-κB and NLRP3 inflammasome pathways generates high levels of inflammatory mediators, such as IL-1β and IL-18 which mediate inflammation-related disorders (20). Therefore, identification of agents, such as SM inhibitors that target these pathways, is a significant step toward developing effective therapeutics for this type of disorder. FMF is a prototypic auto-inflammatory disease characterized by mutations in the C-terminal B30.2 domain of Pyrin. However, it remains controversial as to whether the FMF phenotype is NLRP3-dependent or –independent, and also whether FMF is caused by the loss of an inflammation inhibitor or the gain of a pro-inflammatory molecule’s activity. Hence, the impact of these variants of mutations on Pyrin functions remains unknown (21).

In the current study, for the first time, we evaluated the anti-inflammatory effects of DMAPT, as an SM inhibitor, on the NLRP3-dependent pathway affecting LPS/ATP-stimulated PBMCs of FMF patients. According to the results obtained from inhibition of gene expression by DMAPT, it was found that DMAPT could decrease the expression of genes involved in the production of inflammatory mediators, such as NF-κB, NLRP3, MEFV, CASP1, and IL-1β, followed by a drop in production of IL-1β and IL-18 as inflammatory cytokines in comparison with untreated cells. The effect of DMAPT on the inflammatory process was similarly observed for colchicine. 

Moreover, it was shown that NF-κB was the direct upstream transcriptional activator of NLRP3, MEFV, and CASP1 promoter (8), and NF-κB acted as a transcriptional factor of RelB (a member of the NFκB family), which plays a key role in immune response mediation (22). As reported by Chae *et al*., Pyrin is cleaved by Caspase-1 which interacts with the p65 subunit of NF-κB and IkappaB-alpha and, finally, activates NF-κB. So, they suggested a new Pyrin/Caspase1 pathway for NF-κB activation (23).

Regarding the anti-inflammatory effects of PTL and its compounds, Christine Juliana *et al.* have reported that PTL is a direct inhibitor of NF-κB, Caspase-1, and NLRP3 (20). Also, another study showed that PTL and DMAPT could block the NF-κB pathway in chronic myeloid leukemia (CML) cells by inhibition of p65 phosphorylation (24). Taken together, based on the results of this study as well as previously reported results, it was concluded that DMAPT could inhibit NF-κB at both transcription and protein levels. 

To date, no study has reported the effect of DMAPT on MEFV gene expression or Pyrin protein activation. A study reported a significant decrease in the level of IL-1β in the plasma of a DMAPT-treated pancreatic cancer mouse model (25). Zhang *et al*. also demonstrated that PTL inhibited the production of IL-1β in experimental autoimmune neuritis (EAN) rats (26). Moreover, we found that DMAPT decreased the protein level of IL-18 and IL-1β cytokines and suppressed inflammation. Besides, the secretion level of IL-37 as an anti-inflammatory cytokine and a member of the IL-1 family was evaluated, and it was observed that similar to other IL-1 family cytokines (i.e., IL-1β and IL-18), the secretion of *IL-37 *decreased by DMAPT. Therefore, it could be concluded that DMAPT produced the same inhibitory effects on both pro-inflammatory and anti-inflammatory cytokines of the IL-1 family.

Besides the anti-inflammatory nature of DMAPT, most of the research has focused on the anti-cancer effects of PTL and DMAPT (26-28). Guzmanet *et al. *reported that leukemia cells were very sensitive to PTL treatment and showed a strong apoptotic response at PTL concentration of 7.5 µM, while normal hematopoietic cells had the lowest rate of apoptosis at this concentration (28). Researchers reported that PTL and its derivatives exhibited both anti-inﬂammatory and anti-tumorigenic effects and could inhibit NF-κB pathways via blocking upstream factors at the transcription level (29). In 2014, Viennois *et al*. investigated the effect of Intraperitoneal (IP) injection of DMAPT in a murine model of dextran sodium sulfate(DSS)-induced colitis and showed that DMAPT attenuated this chemically induced colitis(30). In another study on splenocytes from *Experimental Autoimmune Encephalomyelitis (EAE*) mice, it was shown that PTL up to 20 µM is non-cytotoxic to cells and also significantly inhibits *Interferon Ɣ*(IFN-Ɣ), Tumour necrosis factor α(TNF-α), IL-17, and IL-6 production(31). In this study, it was shown that DMAPT, at the concentrations of 6.25 to 12.5 µM, produced the lowest toxicity effects on PBMCs of FMF patients; whereas, DMAPT treatment led to strong apoptotic response at the concentrations of 25 to 50 µM. Therefore, it can be suggested that low doses of DMAPT be used for anti-inflammatory effects *in vivo*.

This work may have limitations that would affect the clinical use of DMAPT. On the other hand, we evaluated the anti-inflammatory effects of DMAPT on gene expression of various inflammatory molecules. While evaluating the protein expression of NF-κB, NLRP3, MEFV, and Caspase-1 can also confirm the findings of their gene expression. 

## Conclusion

Given the results of this study and previous research, DMAPT at the low concentrations has shown a dramatic effect on several key sites in the inflammation pathways, such as NF-κB, NLRP3, MEFV, Caspase1, IL-1β, and IL-18 with the lowest cytotoxic effect on cells. So, the future clinical development of DMAPT or its derivatives may result in the expansion of new anti-inflammatory therapeutics for FMF and other auto-inflammatory disorders. Therefore, this SM is prone to be further investigated through pre-clinical and clinical trials as an alternative adjunctive treatment.

## Funding

This study was financially supported by a grant (grant no: 1109) from the Research and Technology Council of Mazandaran University of Medical Sciences, Iran**. **

## Ethical approval code

IR.MAZUMS.REC.1398.079

## Authors’ Contributions

AM, AR, and RSH designed the experiments; AM performed experiments and collected data; AM, AR, RSH, SA and MG discussed the results and strategy; AR Supervised, directed and managed the study; AM, AR, and RSH Final approved of the version to be published. 

## Conflicts of Interest

The authors declare no conflicts of interest.
